# Symbiotic microbiota and odor ensure mating in time for giant pandas

**DOI:** 10.3389/fmicb.2022.1015513

**Published:** 2022-11-17

**Authors:** Rui Ma, Weichao Zheng, Junliang Guo, Rong Hou, He Huang, Fei Xue, Yanshan Zhou, Wei Wu, Chong Huang, Jiang Gu, Feifei Feng, Xiang Yu, Jiabin Liu, Zusheng Li, Long Zhang, Guanwei Lan, Chao Chen, Wenlei Bi, Qiang Dai, Jacob R. Owens, Hong Yang, Xiaodong Gu, Qi-gui Yan, Dunwu Qi

**Affiliations:** ^1^College of Veterinary Medicine, Sichuan Agricultural University, Chengdu, China; ^2^Sichuan Key Laboratory of Conservation Biology for Endangered Wildlife, Chengdu Research Base of Giant Panda Breeding, Chengdu, China; ^3^Sichuan Academy of Giant Panda, Chengdu, China; ^4^Chengdu Institute of Biology, Chinese Academy of Sciences, Chengdu, China; ^5^Los Angeles Zoo & Botanical Gardens, Los Angeles, CA, United States; ^6^Daxiangling Nature Reserve, Yaan, China; ^7^Sichuan Forestry and Grassland Bureau, Chengdu, China

**Keywords:** *Ailuropoda melanoleuca*, reproduction, anogenital gland secretion, microbiota, chemical communication, covary

## Abstract

To achieve reproduction, male solitary mammals need to locate females using chemical communication with high levels of precision. In the case of giant pandas, the total estrus period of females was usually 15 days each year, however, successful mating activity is finished within 3 days from respective home range. The mating pattern of giant pandas, where multiple males compete for each female requires females employ efficient systems to communicate their estrus phases. To verifying whether the scent secretions of giant pandas changes by gender and estrus progression, the microbiota and compounds in 29 anogenital gland samples from 14 individuals during estrus were analyzed by 16S rRNA sequencing and GC-MS. We show that the microbiota communities covary by gender with 4 particular compounds of scent secretions. Among 597 genera, 34 were identified as biomarkers that could be used to distinguish between different estrus phases. By bacterial-compounds co-analysis, 3 fatty ester acids and squalene compounds covaried with the development of estrus in the bacterial communities of female giant pandas. This study helps clarify how a large, solitary mammal expresses accurate information to improve the likelihood of successful reproduction by changing the composition of microbiota and odor compounds of anogenital glands during estrus.

## Introduction

Chemical communication is one of the most important means of communication among mammals, especially in the social behaviors of solitary mammals (Sergiel et al., 2017; Doyle and Meeks, 2018; Scaloni, 2020). Based on the abundances and compositions of the compounds, host scents can convey a wealth of information, including individual physiological characteristics (Echeverri et al., 2021), social position (Theis et al., 2013), estrus status (Silambarasan et al., 2019), territorial ownership (Marneweck et al., 2018), and possession of natural resources (Zu et al., 2020). For example, male American bison (*Bison bison*) can move from a herd of non-estrus females over a fence to a herd of rutting female Bison 3 km away (Berger, 1992). Male Asian elephants (*Elephas maximus linnaeus*) can identify female Asian elephants in the follicular phase or luteal phase through urine to determine their reproductive status (Slade et al., 2003). In the anogenital glands of European badgers (*Meles meles*), 56 bacteria were involved in the production of odor molecules, while in the anogenital glands of Meerkats (*Suricata suricatta*), 251 OTUs were involved in the production of odor molecules (Sin et al., 2012; Leclaire et al., 2014). The microbiota and odors of anogenital glands of Spotted hyenas (*Crocuta Crocuta*) can work together to identify sex, age, pregnancy, and member ship (Theis et al., 2013).

Giant pandas (*Ailuropoda melanoleuca*) have evolved a chemical communication system that uses anogenital gland secretions (AGS) as information carriers to demonstrate their social behaviors (Ellis et al., 2006). By marking scent information on the ground, tree trunks, rocks, and other objects, the giant panda is able to send personal information intended to adjust the behavior of the recipient during social activities, such as establishing a territory and breeding (Yuan et al., 2004; Nie et al., 2012). In total, 39 compounds in the AGS of captive adult giant pandas during the non-mating season and presumed compound composition differences associated with sex and individual identity existed (Zhang et al., 2008). These volatile compounds were present only during the mating season in the AGS of male adult pandas and that the relative abundances of many compounds changed as a function of breeding season, whereas non-volatile compounds were lower in the mating season (Zhou et al., 2019). The diverse community of fermentative bacteria with enzymes that support metabolic pathways for the production of volatile odorants specialized for chemical communication in giant pandas (Zhou et al., 2021). However, the effects of estrus on the AGS odors of giant pandas during estrus season have not yet been investigated.

Giant pandas are considered relatively solitary mammals with average home range estimates of 3.9–6.1 km^2^ (Schaller, 1985). In general, pandas each live in their own home ranges during the non-estrus season, and there are short periods of aggregation during the estrus season (Martin-Wintle et al., 2019). By marking AGS scent secretions, the wind can transmit a female’s information to males several kilometers away. When males receive the message that a female is in estrus, they flock to her location and fight for the first mating order (Martin-Wintle et al., 2019; Zhou et al., 2021). We assume that as solitary animals with a mating mode in which several males compete for individual females, the AGS chemical communication efficiency in female giant pandas is essential to mating success. Furthermore, transmitting accurate and real-time AGS information of a female’s estrus state is critical to sync appropriate and timely mating behaviors among individuals in the local community.

Limited captive conditions may hinder effective chemical communication and lead to reproductive failure in captive pandas (Swaisgood et al., 2004). Due to different living environment, stress, diet and other factors, can lead to individuals hormone levels and symbiotic bacteria composition differences (Lizé et al., 2013; Ezenwa and Williams, 2014). The results of a comparative study of odor compounds in the anogenital glands of male giant pandas in different environments showed that, wild individuals had higher levels of short-chain fatty acid and aldehydes and lower levels of esters and fatty acids in their AGS (Zhou et al., 2019). Unfortunately, the research on chemical communication of anogenital glands in female giant pandas during estrus is still blank. The potential reasons are as follows: (i) the frequency of marking behavior of anogenital glands in females is less in non-estrus period and (ii) the female giant panda has a short estrus period and is full of aggression, which poses a severe challenge to the collection of samples in the wild (Zhou et al., 2019). Therefore, the study of anogenital gland markers in captive female giant pandas is a crucial first step in understanding the chemical communication of female giant pandas, which can provide theoretical basis and technical support for future field research. At the same time, the study of captive individuals can explain the ecological behavior of this species to a certain extent (Kleiman et al., 1997; Zimbler-DeLorenzo and Stone, 2011; Culliney et al., 2012; Bowen et al., 2021).

In this study, the changes and correlations between microbiota communities and compounds of AGS during estrus were analyzed using next-generation sequencing and GC-MS. As such, this study also provides a theoretical explanation for the different information functions of the AGS produced by female giant pandas at different phases of estrus. In addition to the increased understanding of the composition and covaried changes of symbiotic bacterial and odor compounds of AGS in giant pandas provided herein, these results contribute to our understanding and application of *ex situ* conservation efforts.

## Materials and methods

### Subjects

All the samples were collected from 14 adult giant pandas (Male, *n* = 4; Female, *n* = 10; all age > 5 years) housed at the Chengdu Research Base of Giant Panda Breeding, Chengdu, China. All the individuals were kept in traditional enclosures (Zhang et al., 2008). As determined by veterinarian, all individuals were healthy and free of antibiotics for three months prior to sample collection. The information of samples and individuals was shown in Supplementary Table 4.

### Sample collection and extraction

In this study, the AGS used to compare sex-related differences were collected opportunistically directly from the surface of the pandas’ anogenital glands after being anesthetized for semen collection or artificial insemination by veterinarians (as part of the standard protocols for the Chengdu Panda Base). The secretions used to study the differences between female giant pandas at different phases were collected from the environment when an individual rubs (marks) a surface, which are referred to as anogenital glands marker (AGM) below. All the AGS and AGM samples were collected during November 2018 to April 2019 mating season.

For fresh AGS samples, sterilized cotton swabs were used to wipe the sticky black secretions from the inside of the anus while anesthetized. For fresh AGM samples, the keepers used apples to transfer subjects to an adjacent den immediately after pandas marked with anogenital gland secretions. The fresh panda AGM were collected from the surface of tree trunks, wooden bed, cement floor, and cement walls by rubbing the surfaces with sterilized cotton swabs.

In addition, two more swabs were used to wipe the environmental surface in the vicinity of the marker to collect environmental compounds and microbiota for the purpose of correcting the results of AGM analysis to remove background noise caused by the environment. To test the effect of age on the sample quality and, in the case that AGM were not found to decrease in quality between sampling intervals, provide repeated sampling of the microbiota, marked areas were swabbed three additional times at 2, 4, and 6 h after the initial sampling (T-0) that occurred immediately following the observed marking event. To do so, prior to initial swabbing the marked surface was visually divided into four parts and one quarter was swabbed at each time interval.

All samples for odor compounds analysis were packed on ice and all samples for microbiota analysis were packed in dry ice freezer, both shipped to the laboratory of Chengdu Research Base of Giant Panda Breeding. All samples for odor analysis were stored at −20°C and all samples for microbiota analysis were stored at −80°C until analysis. Samples for AGS and AGM odor compound analysis were processed following the methods of (Zhou et al., 2019) without modification. In general, 600 μl dichloromethane (purity > 99.8%, Macklin) was added in each tube with AGS odor compounds analysis sample swab and vortexed for 15s, then stored at −4°C for 12 h. After that, the samples were centrifuged for 3 min at 3,500 r/min and resulting supernatants were transferred to new 2-ml clean centrifuge tubes and stored frozen at −20°C until they were analyzed by GC-MS. During all stages of collection and handling of odor samples, latex gloves and disposable surgical masks were worn to avoid contamination with human scent.

The estrus period in female giant pandas is short and the progressive phases are difficult to classify through observations, and so estrogen and progesterone in the urine of female giant pandas were analyzed to identify the estrus phase of AGM samples collected on a given day from the focal subjects between 15:00 and 20:00 (Kersey et al., 2010). All urine samples were collected at 6:00–8:00 am each day, starting at the observation of estrus-related behaviors and indicators, including loss of appetite, restlessness and frequent bleating, increased walking or water play (Nie et al., 2012), and terminating once mating occurred. Fresh urine samples were collected during urination using a disposable syringe and subsequently injected into a 10-ml centrifuge tube, which was immediately sent on an ice pack to the Chengdu Research Base of Giant Panda Breeding Endocrine Laboratory for estrogen and progesterone tests.

To correlate the content changes of vocal and chemical communication during estrus of giant pandas, vocalizations were recorded. Using Shure VP89M directional microphone and Tascam DR-100MKII handheld recorder (frequency range: 10 Hz–40 kHz, +0.1/−0.5 dB) recorded bleating sounds of the experimental subjects during the all estrus period. The sampling rate of the recorder was set to 48 kHz and 16bit to ensure that high-quality sound parameters were captured and format as WAV files. In order to eliminate the influence of recording distance on experimental data, the collection distance of all recorded vocal was obtained within 2.5 m from the focus individual.

### Estrous status identification

To assess whether the AGM samples were collected during the prophase, metaphase, or anaphase of estrus, urinary values of creatinine, estrogen, and progesterone were analyzed. Before testing, urine samples were gently thawed at 4°C for 1 h to allow the impurities to precipitate. Creatinine (Cr), estrogen, and progesterone values were tested according to the method and procedure reported in (Kersey et al., 2010), all reagents and lab supplies were identical. The estrogen and progesterone concentration in a given urine sample (ng/ml) were divided by Cr concentration (mg/ml) to derive a final hormone concentration that was expressed as the concentration of the hormone per mg Cr excreted (ng/mg Cr). The standard estrous hormonal status of the female giant panda shows certain regular patterns (Hama et al., 2009; Kersey et al., 2010; Lindburg et al., 2010). Estrogen should rise steadily during the prophase, metaphase, and peaks at anaphase, and then immediately declines to the end of estrus (postphase). Progesterone should stay low in the prophase, metaphase, and anaphase of estrus, and rises rapidly at postphase. Subjects in our study with changes in their estrogen and progesterone values that did not follow this pattern were considered unqualified, a criterion that excluded five female pandas from this study.

### Compounds surveys

The AGM odor sample compound results were subtracted from the compounds in the environmental samples collected to obtain the true AGM compound composition for analyses. Among all compounds, those with a relative abundance of less than 0.1% were removed, and those that appeared in three or more different pandas were retained as core compounds of the giant panda anogenital gland during estrus. A Shimadzu Gas Chromatograph Technologies system (GC-2010) equipped with an HP-5ms glass capillary column (0.25 mm i.d. × 0.25 μm film thickness) was used to analyze the compound compositions of the giant panda perianal gland odor samples and environmental samples. A 1 μl sample was injected using the splitless mode and mass scanning was performed using the full-range scanning mode. Helium gas was set to constant flow (1.0 mL/min) and splitless mode was applied. Injection port temperature was set at 280°C, the initial oven temperature was set at 35°C with a retention time of 1 min, followed by an incremental increase in temperature to 280°C at a rate of 10°C/min, and held for 10 min. Electron impact ionization was used at 70eV and the transfer line temperature was 280°C. The entire run lasted 50 min and tests revealed that no compounds eluted after 40 min. Compounds were initially identified by matching their retention time and mass spectra with structures available in NIST 2002 library (Agilent Technologies 2002, USA).

### Bacterial surveys

DNA was extracted from AGS, AGM, and environmental samples using a BIOG Swab DNA Kit (Changzhou Bio-generating Biotechnologies, China) according to the guide protocol. The DNA quality of each sample was determined by 1% agarose gel electrophoresis, and sent to Shanghai Majorbio Bio-pharm Technology Co., Ltd (Shanghai, China) on dry ice for further concentration assessment, amplification, and sequencing according to their standard procedures (Wu et al., 2022; Xu et al., 2022). The raw reads were deposited into the NCBI Sequence Read Archive (SRA) database (Accession Number: PRJNA781343, PRJNA781346, PRJNA781356, and PRJNA781364). The V3–V4 regions of the 16S rRNA gene were amplified using the primers 338F (5′-ACTCCTACGGGAGGCAGCAG-3′) and 806R (5′-GGACTACGCGGGTATCTAAT-3′) that target conserved sequences found in bacteria. PCR reactions were performed in triplicate using 20 μl mixture containing 4 μl of 5× FastPfu Bufer, 2 μl of 2.5 mM dNTPs, 0.8 μl of each primer (5 μM), 0.4 μl of FastPfu Polymerase and 10 ng of template DNA. The amplicons were then extracted from 2% agarose gels and further purified using the AxyPrep DNA Gel Extraction Kit (Axygen Biosciences, Union City, CA, USA) and quantified by QuantiFluor-ST (Promega, USA) according to the protocols. Purified amplicons were pooled in equimolar and paired-end sequenced (2 × 300) on an Illumina MiSeq platform (Illumina, San Diego, USA) according to the instruction. Raw reads were demultiplex and quality-filtered using QIIME (version 1.9.1). The taxonomy of each 16S rRNA gene sequence was analyzed against the SILVA 128/16s bacteria database with confidence threshold of 70%. After sampling each sample to a smallest sequencing depth and clustering, all operational taxonomic units (OTUs) at 97% identity were obtained using UPARSE (version 7.0).

To minimize the interference of environmental microbes with the real microbiome in AGM, the composition differences between the AGS samples and the environmental samples were compared, OTUs present only in the environmental samples from the AGM samples were removed, and the OTUs shared between the AGS and environmental samples were retained to obtain the real AGM microbiota composition.

### Statistical analysis

To indicate changes in estrus status, the difference between estrogen and progesterone values was used to determine the stage of estrus and was calculated using Repeated Measures ANOVA and displayed in Graphpad Prism 9. In order to verify whether AGM samples with background noise removed can be used for subsequent analysis, we conducted the analysis of similarities (ANOSIM) and non-metric multidimensional scaling (nMDS) plots based on Bray–Curtis index analysis of OTUs in environmental samples, AGS samples and AGM samples with noise removed. Because absolute concentration is subject to variability across samples, the relative abundance of each chemical compound within a sample (i.e., proportion) was used for statistical analyzes. The shared and unique OTUs between groups and samples, based on the occurrence of OTUs in a sample group regardless their relative abundance, were visualized using Mothur software (version 1.31.2). The difference of OTUs was shown by Venn using R (3.6.1). Alpha diversity indices and richness calculations of AGS were performed using the Wilcoxon rank-sum test and the Repeated Measures ANOVA test, AGM and collection time quality control AGM samples were performed using the Repeated Measures ANOVA test, all displayed by Graphpad Prism 9. The relative abundance of bacteria at the genus and phylum level, and core odor compounds of AGS and AGM samples was presented using R ggplot2 (3.6.1). The difference of bacteria on genus and phyla level and core odor compounds of AGS was obtained by the Wilcoxon rank-sum test and performed fdr for multiple test correction, those of AGM samples were obtained by the Repeated Measures ANOVA test and also performed fdr for multiple test correction and performed Tukey for the *Post-hoc* test, all shown in Graphpad Prism 9. The identification of bleating sound is the same as described in Charlton’s report (Charlton et al., 2010). According to the spectral and time domain characteristics of bleating sound, the identification of bleating sound is determined by PRAAT (5.3.41) software (Boersma, 2006). The LDA analysis was being used to look for potential biomarkers of sex and estrus phase differences and created using Galaxy online analysis, the differences of different estrus phases were classed according to the estrus phases, and individuals were identified as the subclasses (LDA scroe > 2.0). The second-order polynomial curve fit and spearman correlation was being used for analyzing the trend between female pandas’ estrus dates and bleating frequency, bleating frequency, bacterial diversity, species of AGSMs and fatty acid ester calculated and showed in Graphpad Prism 9. The variation in the OTUs and odor compounds profile similarities of AGS and AGM samples was visualized through 2D, non-metric multidimensional scaling (nMDS) plots based on Bray-Curtis index by R Vegan package (3.6.1). The analysis of similarities (ANOSIM) was used to determine the difference between OTUs and compounds in different sex, a two-way ANOSIM considered individual identify facter was used to determine the difference between OTUs and compounds in different estrus phases, all ANOSIM tests were created using R vegan package. The correlation of compounds with various estrus stages was visualized through 2D, PCA analysis of factor constrained was statistically evaluated using Redundancy Analysis (RDA) when the Detrended Correspondence Analysis (DCA) lengths of gradient value of species-samples were less than 3.5 and using Canonical Correlation Analysis (CCA) when they were not, the r-square and significance was assessed using the Permutest function in the vegan package of R (3.6.1). After accounting individual identity data were taken as the control matrix, the effect of different compounds on the composition of the microbiota in AGM was assessed using the Partial Mantel test and presented using R vegan package. Microbial functions were predicted using PICRUSt2 (version 2.5.0) and aligned to the Kyoto Encyclopedia of Genes and Genomes (KEGG) database. The relative abundance of KEGG pathway on level 2 of sex and estrus phases visualized through heatmap graph by R (3.6.1). The LDA analysis was being used to look for significant difference KEGG pathway on level 2 of sex and estrus phases and created using Galaxy online analysis (LDA > 2.0). In this study, significant differences were considered significant when *p* < 0.05 and extremely significant when *p* < 0.01, and the statistical analysis (Repeated Measures ANOVA and Wilcoxon rank-sum Tests) was applied Software SPSS25.0 (SPSS Inc., Chicago, IL, USA).

## Results

### Anogenital glands marker samples quality assurance

We found no significant changes in the richness (Repeated Measures ANOVA, Sobs, *p* = 0.885; ACE, *p* = 0.470; Chao, *p* = 1.000) or diversity (*p* = 0.885; Simpson, *p* = 0.312) of the microbiota in the AGM samples (Supplementary Figure 1) over time, indicating that the microbiota in AGM is very stable over a period of at least 6 h. All AGM samples in this study were collected within 2 h, which was sufficient to ensure the fidelity of subsequent analyses. The structure of bacterial communities among environmental samples, AGS samples, and AGM samples was significantly different (nMDS; stress = 0.158, ANOSIM; R = 0.494, *p* = 0.001; Supplementary Figure 3). The structure of bacterial communities between environmental samples and AGS samples was significantly different (nMDS; stress = 0.144, ANOSIM; R = 0.694, *p* = 0.001). The structure of bacterial communities between environmental samples and AGM samples also showed significantly different (nMDS; stress = 0.115, ANOSIM; R = 0.941, *p* = 0.001). However, there was no difference between female’s AGS samples and AGM samples in the structure of bacterial communities (nMDS; stress = 0.140, ANOSIM; R = 0.017, *p* = 0.314). Therefore, the AGM samples with background noise removed are of good quality and can support subsequent analysis.

### Hormonal changes provide rationale for anogenital glands marker sample collection

**FIGURE 1:**
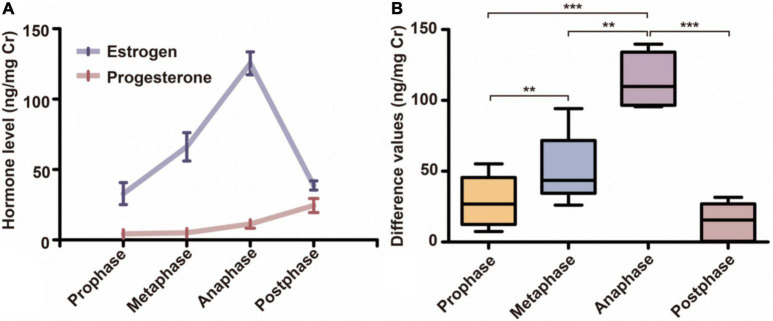
The hormonal changes of female pandas during estrus. **(A)** The variations in urine estrogen and progesterone of female pandas during four different estrus periods (*n* = 20). **(B)** The difference values between urinary estrogen and progesterone in female pandas changed during four different estrus periods (*n* = 20). **p < 0.01; ***p < 0.001.

Based on the standard pattern of female giant panda hormonal changes in estrus, the prophase, metaphase, anaphase, and endphase of estrus were determined when the difference value of urine estrogen and progesterone showed significant differences. After data processing with Cr, the urine estrogen (range from 15.46 to 148.27 ng/mg Cr) and progesterone (range from 1.78 to 40.4 ng/mg Cr) of female pandas (*n* = 5) presented the standard estrus hormonal trends (Figure 1A). The difference values (range from 0 to 128.43 ng/mg Cr) between two adjacent phases all showed significant differences that AGM samples were sufficient to represent different estrus phase (Repeated Measures ANOVA test, prophase vs. metaphase, *p* = 0.002, metaphase vs. anaphase, *p* = 0.006, prophase vs. anaphase, *p* = 0.001, anaphase vs. endphase, *p* = 0.0001, Figure 1B). All AGM samples were collected from pandas anesthetized by veterinarians for insemination (as a standard practice) after the anaphase AGS and AGM had already been collected, which made it difficult to collect AGM samples at endphase because of the effects of anesthesia and the reduction in marking behavior. A total of 15 samples from 5 females were of acceptable quality, equally distributed with prophase, metaphase, and anaphase of estrus.

### Chemical compounds in odor samples of giant pandas during estrus

A total of 120 chemical compounds were identified in the AGS (*N* = 14) samples and AGM (*N* = 15) samples of giant pandas (excluding those also found in the environmental samples), including 36 compounds shared by both sexes, 23 identified only in males, and 61 only in female samples. Of these 120 compounds, 47 were non-volatile (molecular weight >300, 17 shared between sexes, unique: male vs. female = 6 vs. 24) and 73 were volatile compounds (molecular weight < 300, 19 shared between sexes, unique: male vs. female = 17 vs. 37), volatile compounds comprised 60.01% of male samples and 57.73% of female samples (Supplementary Table 1). The identified compounds were comprised of 35 fatty acid esters (shared = 13, unique: male vs. female = 4 vs. 18), 28 alkanes (shared 4, unique: male vs. female = 7 vs. 17), 13 alcohols (shared 4, unique: male vs. female = 2 vs. 7), 11 sterols (shared 4, unique: male vs. female = 1 vs. 6), 6 fatty acid (shared 2, unique: male vs. female = 2 vs. 2), 6 alkane derivatives (shared 1, unique: male vs. female = 2 vs. 3), 4 aldehydes (unique: male vs. female = 1 vs. 3), 3 heterocyclic compounds (shared 1, unique 2 in female), 2 amides (shared), 3 alkenes (shared 2, 1 unique in female), 2 quinones (unique: male vs. female = 1 vs. 1), 1 alkyne (in female), 1 ketone (in female), 1 ether (in male), squalene (shared), and 3 other compounds (shared 1, unique: male vs. female = 1 vs. 1). The AGS chemical profile for each sample consisted of a subset of the 120 identified compounds ranging from 18 to 27 detected per sample.

### The bacterial and compounds’ profiles of male and female are markedly different

**FIGURE 2:**
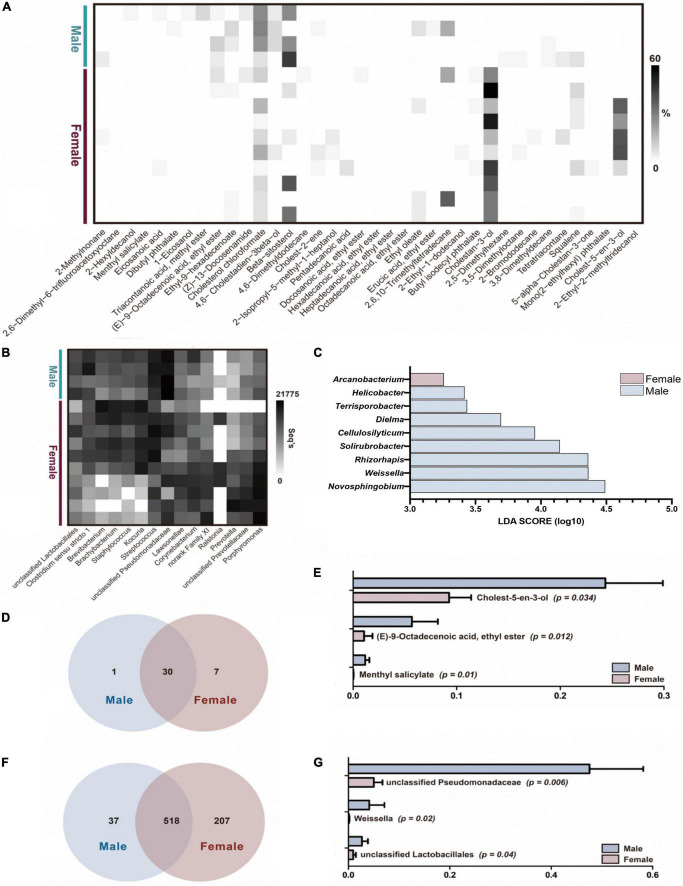
The difference of bacterial and compounds’ profiles between male and female pandas AGS samples. **(A)** The odor core compounds abundance heatmap of AGS between male and female pandas during estrus (*n* = 14). **(B)** The top 15 microbiota community abundance heatmap on genus level of AGS between male and female pandas during estrus (*n* = 14). **(C)** The Liner Discriminant Analysis (LOA) analysis of AGS bacterial profiles between male and female pandas during estrus (The graph shows the LOA scores obtained from linear regression analysis of the significant microorganism groups in the two groups. When the default LOA value is more than 2 and the *p* value is less than 0.05, the result corresponds to a different species, *n* = 14). **(D)** The unique and shared compounds between male and female giant pandas during estrus in AGS core compounds (*n* = 14). **(E)** The significant difference of AGS core compounds between male and female giant. pandas during estrus (*n* = 14). **(F)** The unique and shared OTUs between male and female giant pandas during estrus (*n* = 14). **(G)** The significant difference of AGM top 20 bacteria on genus level between male and female giant pandas during estrus (*n* = 14).

Compounds that are referred to as AGS core compounds, which included 38 compounds and comprised 14 fatty acid esters, 6 sterols, 7 alkanes, 5 alcohols, 2 fatty acids, 1 alkane derivatives, 1 amide, 1 alkene, and 1 squalene (Figure 2A). Overall, of the 38 core compounds, males and females share 30 common compounds, with one unique compound, dibutyl phthalate, for male giant pandas and seven unique compounds, 2-ethyl-1-dodecanol, butyl isodecyl phthalate, cholestan-3-ol, 5-alpha-cholestan-3-one, mono(2-ethylhexyl) phthalate, cholest-5-en-3-ol and 2-ethyl-2-methyltridecanol, for female giant pandas (Figure 2D). In the shared compounds, cholesterol chlorofomate (Wilcoxon sun-rank test, *p* = 0.034), (E)-9-octadecenoic acid ethyl ester (*p* = 0.012), and methyl salicylate (*p* = 0.01) were significant enriched in male giant panda AGS (Figure 2E). However, no odor compound was found in AGS that could be used as a biomarker to distinguish by LDA analysis between the sexes of estrus giant pandas.

A total of 647,467 sequences were generated from 14 AGS odor samples, with an average of 46,248 sequences per sample before clustering (ranging from 36,959 to 65,922). The average sequence length was 420 bp, with the maximum length being 516 bp and the shortest length being 210 bp. After sampling each sample to an equal sequencing depth, all high-quality sequences at 97% identity resulted in a total of 20 phyla, 34 classes, 76 orders, 158 families, 362 genera, 547 species, and 762 OTUs. The male group had higher bacterial diversity and richness but no significant differences with those of females (Table 1A). Both genders shared 518 OTUs, 37 unique OTUs were sequenced in male group and 207 unique OTUs were sequenced in female group (Figure 2F). The most abundant phyla (relative abundance >1% of all samples) were Firmicutes (totally 30.29%, male vs. female = 34.85% vs. 28.46%), Actinobacteria (totally 26.67%, male vs. female = 11.28% vs. 32.83%), Proteobacteria (totally 23.34%, male vs. female = 51.91% vs. 11.92%), and Bacteroidetes (totally 18.74%, male vs. female = 1.43% vs. 25.67%). Among the dominant phyla, Proteobacteria was significantly enriched in male AGS samples (Willcoxon rank-sum test, *p* = 0.009) and Bacteroidetes was significantly enriched in female AGS samples (*p* = 0.028). The relative abundance of the top 15 most dominant genera range from 6,759 seqs to 75,596 seqs (Figure 2B). Among the top 15 bacteria genus, unclassified Pseudomonadaceae (Willcoxon rank-sum test, *p* = 0.006), Weissella (*p* = 0.02), and unclassified Lactobacillales (*p* = 0.04) were enriched in male group (Figure 2G).

**TABLE 1:** Alpha diversity among AGS and AGM samples of giant pandas during estrus.

Sample group	Sample size	Sobs (Mean ± SD)	Ace (Mean ± SD)	Chao (Mean ± SD)	Shannon (Mean ± SD)	Simpson (Mean ± SD)	Coverage (Mean ± SD)
**A. Alpha diversity among AGS microbiota of estrus giant pandas**							
Male	4	314.25 ± 83.84	446.46 ± 68.9	426.8 ± 104.75	2.44 ± 0.54	0.19 ± 0.07	0.99
Female	10	269 ± 151.8	343.46 ± 178.11	334.76 ± 182.65	2.93 ± 0.59	0.13 ± 0.07	0.99
*p* - Value		0.72	0.62	0.52	0.18	0.36	
**B. Alpha diversity among AGM microbiota of female giant pandas with different estrus period**							
Prophase	5	501 ± 225.61	679.18 ± 269.14	655.41 ± 250.59	3.53 ± 0.73	0.08 ± 0.05	0.99
Metaphase	5	478 ± 145.52	639.07 ± 235.13	631.07 ± 231.76	3.18 ± 0.42	0.13 ± 0.09	0.99
Anaphase	5	224.8 ± 186.73	351.95 ± 274.48	308.91 ± 263.24	2.54 ± 0.54	0.16 ± 0.05	0.99
*p* - Value		0.09	0.09	0.006	0.04	0.06	

The results of the LDA (Figure 2C) showed that the microorganisms that were significantly different in the AGS from male giant pandas belonged to *Helicobacter*, *Terrisporobacter*, *Dielma*, *Cellulosilyticum*, *Solirubrobacter*, *Rhizorhapis*, *Weissella*, and *Novosphingobium* (Figure 2C). Moreover, the results showed that those were significantly different in the AGS from female giant pandas belonged to *Arcanobacterium* (Figure 2C). The relative abundance of these difference genera was showed in Supplementary Table 2. Those different microorganisms could be used as AGS biomarkers to distinguish between genders of pandas during estrus (LDA > 2, *p* < 0.05).

The functional genes of the samples were analyzed using the KEGG database. A total of 46 KEGG pathways on level 2 were detected in the AGS samples. The male and female groups shared the same top five KEGG pathways on level 2 with little difference in overall relative abundance. The most dominant pathways of samples were Global and overview maps (totally 39.86%, male vs. female = 39.16% vs. 40.55%), carbohydrate metabolism (totally 9.29%, male vs. female = 8.77% vs. 9.81%), amino acid metabolism (totally 7.50%, male vs. female = 7.56% vs. 7.43%), metabolism of cofactors and vitamins (totally 4.48%, male vs. female = 4.37% vs. 4.58%), and energy metabolism (totally 4.22%, male vs. female = 4.22% vs. 4.21%; Supplementary Figure 2A). The estimated cumulative relative abundance of these five dominant pathways was above 60% of the identified KEGG pathways on level 2. The LDA results showed that there was a significant difference between the pathways on KEGG level 2 in the AGS samples from the male group and the female group (Supplementary Figure 2C). There were 14 pathways on KEGG level 2 enriched in male AGS samples, including Cell motility, Signal transduction, Cellular community prokaryotes, Development and regeneration, Drug resistance antimicrobial and other 9 pathways (LDA > 2, *p* < 0.05). There were 6 pathways on KEGG level 2 enriched in female AGS samples, including Global and overview maps, Nucleotide metabolism, Immune disease, Endocrine system, Transport and catabolism and Aging (LDA > 2, *p* < 0.05). Our results showed that the pathways on in male AGS samples are different with those in female AGS samples on KEGG level 2.

### The bacterial and compounds’ profiles of male and female covary

**FIGURE 3:**
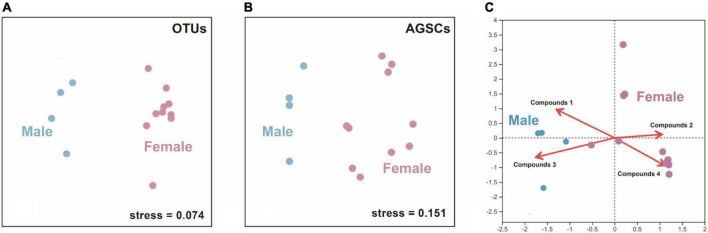
The differences and correlation between male and female estrus pandas in the microflora (OTUs) and anogenital gland secretion compounds (AGSCs). **(A)** A non-metric multidimensional scaling (nMDS) plot showing a difference in structure (Bray–Curtis index) between the AGS bacterial communities of adult male and female giant pandas in estrus (*n* = 14). **(B)** An nMDS plot showing a difference in structure between the AGSCs profiles of adult male and female giant pandas in estrus (*n* = 14). **(C)** A canonical correlation analysis (CCA) plot showing a correlation to the AGS compounds and AGS bacterial communities of different sex. (*n* = 14). Compounds 1–4 represent: (E)-9-octadecenoic acid ethyl ester, cholestane-3-ol, dibutyl phthalate and 2-isopropyl-5-methyl-1-heptanol, respectively.

The structure of bacterial communities in AGS between male and female giant pandas in estrus was significantly different (ANOSIM; male vs. female: R = 0.438, *p* = 0.015; Figure 3A). The AGS of adult male and female giant pandas during estrus had significantly different compound profiles (ANOSIM; male vs. female: R = 0.681, *p* = 0.005; Figure 3B). There was no covariance between the OTUs—core compounds correlation matrices of adult male and female giant pandas during estrus (correlation r, Mantel test, R = 0.108, *p* = 0.314). However, canonical correlation analysis (CCA) plot (detrended correspondence analysis (DCA) axis lengths = 4.109) showed the profiles of (E)-9-octadecenoic acid ethyl ester (Pearson, R = 0.731, *p* = 0.001) and dibutyl phthalate (R = 0.847, *p* = 0.002) had a positive correlation to the AGS bacterial communities of male giant pandas, cholestane-3-ol (R = 0.726, *p* = 0.001) and 2-isopropyl-5-methyl-1-heptanol (R = 0.763, *p* = 0.001) had a positive correlation to the AGS bacterial communities of females (Figure 3C). Furthermore, the OTUs-specific AGSCs correlation matrices for male and female covaried (correlation r, Mantel test, R = 0.211, *p* = 0.039), indicating that the relative abundance of OTUs correlated with the relative abundance of some specific compounds in similar ways in the AGS of giant pandas during estrus from the different sexes.

### The bacterial and compound profiles of female pandas during different estrus phases are markedly different

**FIGURE 4:**
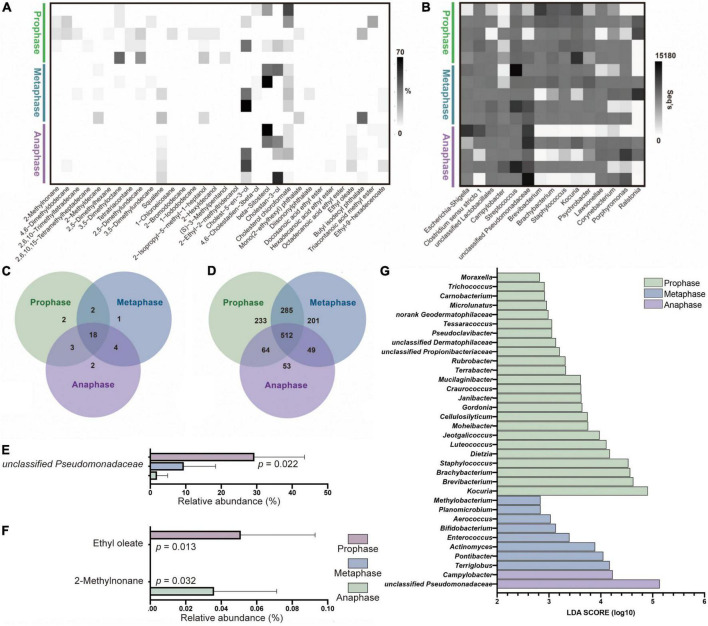
The difference of bacterial and compounds’ profiles between different estrus phases of female panda AGM samples. **(A)** The odor core compounds abundance heatmap of AGM between prophase, metaphase and anaphase of female pandas during estrus (*n* = 15). **(B)** The top 15 microflora community abundance heatmap on genus level of AGM between prophase, metaphase and anaphase of female pandas during estrus (*n* = 15). **(C)** The unique and shared compounds between female giant pandas during prophase, metaphase and anaphase of estrus in AGM core compounds (*n* = 15). **(D)** The unique and shared OTUs between female giant pandas during prophase, metaphase and anaphase of estrus (*n* = 15). **(E)** The significant difference of AGM top 20 bacteria on genus level between prophase, metaphase and anaphase of female giant pandas during estrus (*n* = 15). **(F)** The significant difference of AGM core compounds between prophase, metaphase and anaphase of female giant pandas during estrus (*n* = 15). **(G)** The Liner Discriminant Analysis (LOA) analysis of AGM bacterial profiles between prophase, metaphase and anaphase stage of female pandas during estrus (The graph shows the LOA scores obtained from linear regression analysis of the significant microorganism groups in the three groups, n = 15. When the default LOA value ismore than 2 and the *p* value is less than 0.05, the result corresponds to a differential species).

Similarly, a total of 32 AGM core compounds, including 10 alkanes, 9 fatty acid esters, 5 sterols, 4 alcohols, 3 alkanes derivatives, and 1 squalene, were obtained using the method described above (Figure 4A). The three phases of estrus shared 18 compounds, 2 unique compounds in prophase: 2-methylnonane and 2,5-dimethylhexane, 1 unique compound in metaphase: 2-methyldecane, and 2 unique compounds in anaphase: octadecanoic acid ethyl ester and ethyl oleate (Figure 4C). In all 32 core compounds, 2-methylnonane (Repeated Measures ANOVA test, *p* = 0.032) was enriched in prophase and ethyl oleate (*p* = 0.013) was enriched in anaphase (Figure 4F). However, no odor compound was found in AGM that could be used as a biomarker to distinguish between different estrus phases of female giant pandas.

A total of 1,020,328 sequences were obtained from AGM samples in the three different estrus phases and environmental control samples, with an average of 44,362 sequences per sample before clustering (ranging from 33,021 to 58,239). The average sequence length was 420 bp, with the maximum length being 516 bp and the shortest length being 200 bp. Using an OTU definition of 97% homologous nucleotide base similarity, all sequences resulted in a total of 29 phyla, 55 classes, 126 orders, 240 families, 597 genera, 907 species, and 1397 OTUs after removal of background noise from the environmental control samples (resulting in the elimination of 108 OTUs) and sampling each sample to an equal sequencing depth. At an OTU level, 512 of 1397 OTUs were shared between the AGM of female giant pandas in the three different estrus periods, while the number of unique OTUs decreased with the progression of estrus phase (prophase vs. metaphase vs. anaphase = 233 vs. 201 vs. 53, Figure 4D). Similarly, alpha diversity results show that AGM microbiota richness and diversity significantly decreased, indicating that the microbiota became more specialized as the estrous phases progresses (Table 1B, Repeated Measures test, Chao, *p* = 0.006, Shannon, *p* = 0.04). The phyla with a relative abundance >1% were Proteobacteria (30.33%, prophase vs. metaphase vs. anaphase = 17.37% vs. 29.31% vs. 45.4%), Actinobacteria (29.8%, prophase vs. metaphase vs. anaphase = 50.55% vs. 23.43% vs. 15.41%), Firmicutes (28.56%, prophase vs. metaphase vs. anaphase = 23.86% vs. 36.53% vs. 25.28%), and Bacteroidetes (8.94%, prophase vs. metaphase vs. anaphase = 4.55% vs. 8.89% vs. 13.38%). No bacteria on phyla level showed significant difference among three phases by the Repeated Measures ANOVA test. The relative abundance of the top 15 dominant genera range from 6,836 seqs to 58,662 seqs (Figure 4B). Of the top 15 most abundant genera, only unclassified Pseudomonadaceae (Repeated Measures ANOVA test, *p* = 0.022) was significantly enriched in anaphase (Figure 4E).

We also found significant differences in the community compositions among those three different estrus stage groups. A total of 24 genera, including *Moraxella* and *Trichococcus*, exhibited a relatively higher abundance in prophase group (Figure 4G). 8 genera *including Methylobacterium* and *Planomicrobium* were relatively more abundant in metaphase group. *Campylobacter* and unclassified Pseudomonadaceae were significantly enriched in the anaphase group. The relative abundance of these difference genera was showed in Supplementary Table 3. These differentially abundant taxa can be considered as potential biomarkers (LDA > 2, *p* < 0.05).

Also the functional genes of the samples were analyzed using the KEGG database. A total of 46 pathways on KEGG level 2 were detected in the AGM samples. The different estrus phases groups shared the same top five pathways on KEGG level 2 with little difference in overall relative abundance. The most dominant pathways of samples were Global and overview maps (totally 39.92%, prophase vs. metaphase vs. anaphase = 41.01% vs. 39.84% vs. 38.92%), Carbohydrate metabolism (totally 9.52%, prophase vs. metaphase vs. anaphase = 9.88% vs. 9.65% vs. 9.02%), Amino acid metabolism (totally 7.68%, prophase vs. metaphase vs. anaphase = 8.15% vs. 7.52% vs. 7.39%), Energy metabolism (totally 4.19%, prophase vs. metaphase vs. anaphase = 4.21% vs. 4.14% vs. 4.24%), and Metabolism of cofactors and vitamins (totally 4.16%, prophase vs. metaphase vs. anaphase = 4.07% vs. 3.96% vs. 4.44%; Supplementary Figure 2B). The estimated cumulative relative abundance of these five dominant pathways was above 60% of the identified KEGG pathways on level 2. The LDA results showed that there was a significant difference between the pathways on KEGG level 2 in the AGM samples from different estrus phase groups (Supplementary Figure 2D). There were 4 pathways on KEGG level 2 enriched in AGM samples of prophase including Global and overview maps, Amino acid metabolism, Excretory system, and Xenobiotics biodegradation and metabolism (LDA > 2, *p* < 0.05). There was only 1 pathway on KEGG level 2 enriched in AGM samples of metaphase: Digestive system (LDA > 2, *p* < 0.05). There were 10 pathways on KEGG level 2 enriched in AGM samples of anaphase, including Cell mobility, Development and regeneration, Signal transduction, Glycan biosynthesis and metabolism, and other 6 pathways (LDA > 2, *p* < 0.05; Supplementary Figure 2D). Our results showed that the pathways on in male AGS samples are different with those in female AGS samples on KEGG level 2.

### The bacterial diversity and compound profiles of female pandas during different estrus phases covaried with bleating sound

**FIGURE 5:**
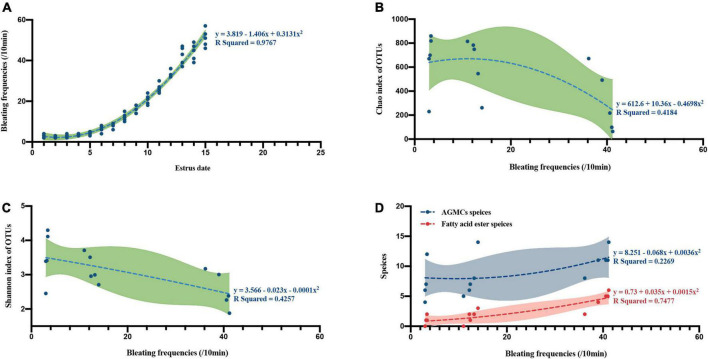
The curve fit and correlation between bleating frequencies and different estrus phases, microflora richness, microflora diversity and different anogenital gland secretion compounds (AGMCs or fatty acid ester). **(A)** A curve fit between estrus date and bleating frequencies (*n* = 5). **(B)** A curve fit between bleating frequencies and microflora Chao richness index (*n* = 15). **(C)** A curve fit between bleating frequencies and microflora Shannon diversity index (*n* = 15). **(D)** Curve fit between bleating frequencies and anogenital gland secretion compounds species (blue stands for AGMCs species, red stands for fatty acid ester species, *n* = 15).

By analyzing the bleating frequency of female giant pandas in estrus on different dates, we found that the bleating frequency increases with the progress of estrus stage (spearman correlation r = 0.9964, *p* = 0.0001; Second-order polynomial curve fit, R squared = 0.9767, Figure 5A). A further correlation and curve fit analysis between bleating frequency and bacterial alpha diversity showed that bleating frequency was negatively correlated with microbial richness (spearman correlation *r* = −0.5921, *p* = 0.0222; Second-order polynomial curve fit, R squared = 0.4184, Figure 5B) and diversity (spearman correlation *r* = −0.6243, *p* = 0.0148; Second-order polynomial curve fit, R squared = 0.4257, Figure 5C). In contrast to the microbiota files correlation results, the species of AGMCs (spearman correlation *r* = 0.5726, *p* = 0.0276; Second-order polynomial curve fit, R squared = 0.2269, Figure 5D) and fatty acid ester (spearman correlation *r* = 0.8982, *p* = 0.0001; Second-order polynomial curve fit, R squared = 0.7477, Figure 5D) were strongly positively correlated with bleating frequency.

### The bacterial and compound profiles of female pandas during different estrus phases covary

**FIGURE 6:**
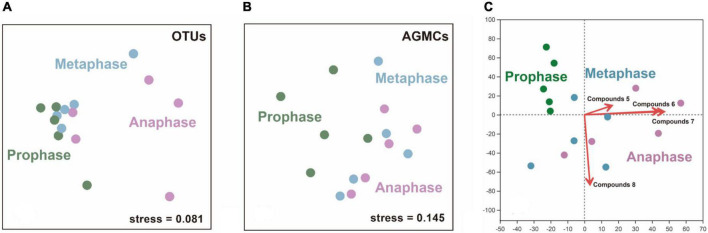
The differences and correlation between different estrus phases of adult female pandas in the microflora (OTUs) and anogenital gland secretion compounds (AGMCs). **(A)** An nMDS plot showing a difference in structure between the AGM bacterial communities of adult female giant pandas at prophase, metaphase and anaphase of estrus (*n* = 15). **(B)** An nMDS plot showing a difference in structure between the AGMCs profiles of adult female giant pandas at prophase, metaphase and anaphase of estrus (*n* = 15). **(C)** A redundancy analysis (RDA) plot showing a correlation to the AGM compounds and AGM bacterial communities of different estrus phase (*n* = 15). Compounds 5-8 represent: hexadecanoic acid ethyl ester, ethyl 9-hexadecenoate, ethyl oleate and squalene, respectively.

The structure of microbiota in AGM of adult female giant pandas during each estrus phase differed significantly from each other and there was a modest effect of individual differences on the structure of bacterial communities (two-way ANOSIM; individual differences: R = 0.253, *p* = 0.200; estrus phases: R = 0.355, *p* = 0.005; Figure 6A). By comparing each phase coupling separately, we found there was only a tendency (no significant difference) for the bacterial profiles to differ between prophase and metaphase (two-way ANOSIM; individual differences: R = 0.452, *p* = 0.221; estrus phases: R = 0.149, *p* = 0.061). But there was a significant difference in the bacterial community structures of AGM between the metaphase and anaphase (two-way ANOSIM; individual differences: R = 0.157, *p* = 0.252; estrus phases: R = 0.611, *p* = 0.005), and also a significant difference was shown between the prophase and anaphase (two-way ANOSIM; individual differences: R = 0.228, *p* = 0.250; estrus phases: R = 0.396, *p* = 0.001). In the composition of the core compounds of AGM, there were a significant differences as the phases of estrus progresses with a modest effect of individual differences on the structure of compounds’ communities (two-way ANOSIM; individual differences: R = 0.195, *p* = 0.052; estrus phases: R = 0.419, *p* = 0.022), which was a consistent finding between prophase and metaphase (two-way ANOSIM; individual differences: R = 0.561, *p* = 0.340; estrus phases: R = 0.179, *p* = 0.116) and between metaphase and anaphase (two-way ANOSIM; individual differences: R = 0.158, *p* = 0.230; estrus phases: R = 0.658, *p* = 0.113). But there was a significant difference between prophase and anaphase (two-way ANOSIM; individual differences: R = 0.423, *p* = 0.298; estrus phases: R = 0.332, *p* = 0.011; Figure 6B), indicating that as the estrus phases progress, the variation of microbiota compositions of AGM was stronger and more obvious than those in the compounds of AGM. Besides, the OTUs—core compounds correlation matrices of all phases in female giant pandas during estrus showed no covariance (correlation *r*, Partial Mantel test, R = 0.066, *p* = 0.271). The redundancy analysis (RDA) plot [detrended correspondence analysis (DCA) axis lengths = 2.997)] showed the profiles of hexadecanoic acid ethyl ester (Pearson, R = 0.327, *p* = 0.045), ethyl 9-hexadecenoate (R = 0.805, *p* = 0.001), ethyl oleate (R = 0.826, *p* = 0.002), and squalene (R = 0.394, *p* = 0.045) had a positive correlation to the development of female giant panda estrus AGM bacterial communities (Figure 6C). In additional, the OTUs-specific AGMCs correlation matrices for estrus phase progression covaried (correlation *r*, Partial Mantel test, R = 0.202, *p* = 0.027), indicating that the relative abundance of OTUs correlated with the percent abundance of some specific compounds in similar ways in the AGM as the estrus phases of female giant pandas progressed.

## Discussion

As a solitary mammal, the giant panda relies on AGS markers for its social communication. In this study, we tested the chemical communication in captive giant pandas during estrus and showed that (i) the bacterial and compounds of giant pandas covaried, (ii) these profiles differed between male and female, and (iii) they varied as female pandas progressed through the stages of estrus.

For female pandas, the period of estrus is basically divided into three phases in the current available research (Wu et al., 2020). In this study, to better investigate the continuous changes in marker composition, a new monitoring stage, defined as the metaphase, was added between the first two phases. Due to the effects of anesthesia or hormonal changes due to conception, marking behavior was greatly reduced making it not possible to collect suitable marker samples at the end of estrus. Thus, the present study focused on changes in AGM in female giant pandas from the start of estrus to the climax of estrus.

Lots of studies have shown that the composition of odor compounds in perianal gland secretions is related to sex in mammals. Male Red-ruffed lemurs (*Varecia variegata rubra*) had significantly different AGS odor compounds than female red-ruffed individuals (Janda et al., 2019). The composition of microbiota and odor compounds in the perianal gland secretion of Spotted hyena (*Crocuta Crocuta*) were different and covaried (Theis et al., 2013). The microbiota and compound profiles were significantly different between male and female pandas in our study. Furthermore, the OTUs-specific compounds correlation matrices for male and female covaried, which also occurred between the different estrous stages in females. This indicates that changes in microbiota structure do not affect the composition of all compounds, but rather convey different information by changing the species and abundance of some specific compounds, which agrees with previous research in laboratory mouse urine-marks (SimeoneZomer et al., 2009). These specific compounds are not just volatile fatty acids but also include some sterols and fatty acid esters, which corresponds to the general symbiotic hypothesis for animal chemical communication (Whittaker et al., 2016; Drea, 2020; Janssenswillen et al., 2021; Mazorra-Alonso et al., 2021).

The anogenital gland secretion microbiota of male and female pandas differs considerably, and this may be related to sex hormones (Miyazaki et al., 2018; Sugianto et al., 2019; Elwell et al., 2021; Poirier et al., 2021). It is well established that steroid hormones are present in mammalian sebaceous and apocrine glands and that they affect gland morphology, production, and chemistry (Comizzoli et al., 2021; Carlitz et al., 2022; Jiang et al., 2022; Peckre et al., 2022). As a steroid hormone, Cholesterol chlorofomate is present with varying abundances in the anogenital gland odor of all giant pandas and presumed to be related to individual identification or estrus status. It has been reported that the urine-marks of giant pandas contains gender and kinship recognition functions (Liu et al., 2008; Dehnhard et al., 2016). Dibutyl phthalate and Cholestan-3-ol, which only obtain in AGS of male and female giant pandas, respectively, may be used as a basis for gender identification (Liu et al., 2008; Dehnhard et al., 2016). Previous studies on the secretions of the giant panda’s perianal glands have shown that males have a higher proportion of volatile compounds in their scent glands (Zhou et al., 2019, 2021). Similar results have been confirmed by chemical communication studies in other animals, such as Owl monkeys (*Aotus nancymaa*e), Tamarins (*Saguinus spp*), Meerkats (*Suricata suricatta*), Otter (*Lutra lutra*), and Spotted hyaena (*Crocuta crocuta*) (Kean et al., 2011; Theis et al., 2013; Leclaire et al., 2017; Bowen et al., 2021; Poirier et al., 2021). Male giant pandas in this study contained a higher proportion of volatile compounds in AGS, which fits with the courtship strategies during the mating season. Compared to females, males have a larger range of activities and during estrus they expand their range in order to find suitable females for matting (Wei et al., 2020). The male panda marker acts as a “flyer”, expressing competitiveness to nearby pandas of the same sex while attracting pandas of the opposite sex. In contrast, the female panda’s marker acts as a “recruitment” during the estrus period, informing nearby males of their estrus status (Ezenwa and Williams, 2014). Considering, fermentation communication is the basis of chemical communication which combine compounds and microbiota of odor sections, the species and abundance of microbiota in the AGS of giant pandas are similar to those of other mammals, suggesting that the anogenital gland secretion microbiota existence of the same fermentation communication mechanism between mammals (Chen et al., 2020; Rojas et al., 2020; Koide, 2022; Kücklich et al., 2022).

The findings of this study are consistent with previous reports: influenced by changes in estrogen and progesterone levels in female pandas during estrus, the abundance of sterols and fatty acid esters increases, while the abundance of alkanes and alcohols decreases as estrus progresses (Meter et al., 2008). Also, the non-volatile high molecular weight compounds were significantly enriched in the anaphase, which typically do not play a direct role in chemical communication. Chemical communication studies of Guinea pigs (*Cavia procellus*), marmosets (*Saguinus Fusciollis*) and Liolaemus wiegmannii lizards (*Iguania liolaemidae*) all showed that the content of squalene increased significantly in odor glands during estrus (Preti et al., 1977; García-Roa et al., 2016). Squalene has been proven to retard evaporation of volatiles and prolong signal persistence by binding compounds, such as carboxylic acids (Greene et al., 2016). Non-volatile high molecular weight compounds such as sterol, ethyl oleate, and octadecanoic acid ethyl ester may enhance the functionality of other semiochemicals and slow release of volatiles (Zhou et al., 2019). Therefore, we postulate that a decrease in hydrocarbons and alcohols in giant panda perianal odor compounds combined with increases in sterol, volatile fatty acid and ester convey information regarding their desire or acceptance of potential mates and estrous stage with different gender and age. In the microbiota profile, unclassified Pseudomonadaceae increased in anaphase, while in anaphase the diversity and richness of the microbiota decrease significantly. As specialist aerobic bacteria, some species of *Pseudomonas* (e.g., *Pseudomonas A5*, *Pseudomonas Su5-2*, and *Pseudomonas SJTD-1*) can metabolized long-chain hydrocarbons to produce short-chain hydrocarbons which may provide the basis for the production of other compounds (Meng et al., 2013). The covariance of microbiota and compounds indicates that the microbiota contributed to the communication of different information (Maraci et al., 2018; Grosser et al., 2019). In this study, genera were identified as biomarkers that could be used to distinguish between gender and different estrus phases. However, no compound was found that could be used as a biomarker. These results show that giant panda chemical communication is not determined by any single compound, but the result of ordered combinations of many compounds.

The progression from one estrous phase to the next has a marked effect on the bacterial and compounds profiles in the anogenital glands of female giant pandas. However, the differences between the prophase-metaphase and metaphase-anaphase are not significant, which indicates that the bacterial and compound profiles are a continually progressing and covariable process. The function of AGS in the prophase is to provide geographical location information and estrus status to male pandas. In the anaphase, AGS changes to convey the most suitable mating time, while the metaphase represents the transition period of progressive estrus status. In this study, estrus phases covaried in symbiotic microbiota and odor compounds to give AGS the ability to convey different message, which is aligned with the mating strategies of giant pandas.

Bleating is considered to be one of the important characteristics of sound communication in giant panda estrus (Charlton et al., 2010). In this study, the richness and diversity of microbiota in AGS decreased with the increase of bleating frequency, showing the specificity and special functionalization of bacterial community structure (Zhou et al., 2019). In addition, the frequency of bleating increased synergistically with the species of compounds and fatty acid esters, suggesting that the role of fatty acid esters in chemical communication is similar to that of bleating in vocal communication, but with its own advantages (Cinková and Policht, 2015; Penteriani and Delgado, 2017). Vocal communication can send a wealth of information at a closer range than chemicals and provide timely and appropriate feedback to the received information, yet it is constrained by the distance of transmission and is relatively short in duration. Chemical communication has the advantage of transmitting information over long periods of time, which can complement the shortcomings of vocal communication.

Giant pandas are in estrus for a short period of time and progress through stages quickly. Male giant pandas need to identify suitable mating partners in time, while the female giant pandas need to show their estrus state in a very short period (2–3 days). As such, effective and timely communication is especially important. In this study, we found that the composition of symbiotic bacteria and odor compounds in giant panda AGS covaried regularly within a short period of time, and demonstrated varying functions at different estrus stages, providing evidence for the complex mating strategies evolved in giant pandas to compensate their solitary behaviors and large ranges. Future research should focus on the correlation and the mechanisms of interaction in anogenital gland microbiota and compounds in order to improve the effectiveness of chemical communication through rational husbandry and management policy.

## Data availability statement

The datasets presented in this study can be found in online repositories. The names of the repository/repositories and accession number(s) can be found in the article/Supplementary material.

## Ethics statement

The animal study was reviewed and approved by the Institutional Animal Care and Use Committee of the Chengdu Research Base of Giant Panda Breeding (IACUC No. 201806).

## Author contributions

Q-gY and DQ conceived and supervised the project. RM, WZ, JG, RH, HH, FX, YZ, WW, CH, JG, FF, XY, JL, ZL, LZ, GL, CC, and WB performed the sample collection. RM, WZ, and JG performed the experiment. RM, WZ, JG, QD, JO, Q-gY, HY, and XG performed the data analyses. RM wrote the manuscript with input from Q-gY and DQ. All authors read and approved the final manuscript.
