# Supplementary material for: Symbiotic microbiota and odor ensure mating in time for giant pandas
Source: Front Microbiol. 2022 Nov 17;13:1015513. doi: 10.3389/fmicb.2022.1015513 (PMC9712809; doi:10.3389/fmicb.2022.1015513)
Supplement: Supplementary file 1 [file Table_1.DOCX]

Table S1 Tentatively identified compounds found in anogenital gland secretion (AGS) in giant pandas during estrus.

| Peck No | Tentatively identified compounds | Formula | Molecular weight | R.Time |  |
| --- | --- | --- | --- | --- | --- |
| 1 | *2,5-Dimethylhexane | C8H18 | 114 | 3.925 |  |
| 2 | 3-Ethyl-2,4-Dimethylpentane | C9H20 | 128 | 4.667 |  |
| 3 | 3-methylbutanoic acid | C5H10O2 | 102 | 5.198 |  |
| 4 | *p-Benzoquinone | C6H4O2 | 108 | 6.514 |  |
| 5 | 5,6-Dimethylundecane | C13H28 | 184 | 6.949 |  |
| 6 | 2,7-Dimethyloctane | C10H22 | 142 | 6.986 |  |
| 7 | 2,4,6-Trimethyloctane | C11H24 | 154 | 7.247 |  |
| 8 | 2,2,3,3-Tetramethylpentane | C9H20 | 128 | 7.260 |  |
| 9 | 2-Methylnonane | C10H22 | 142 | 7.333 |  |
| 10 | (S)-3-Methylpentanol | C6H14O | 102 | 8.725 |  |
| 11 | Nonanal | C9H18O | 142 | 9.769 |  |
| 12 | 3,5-Dimethylundecane | C13H28 | 184 | 10.050 |  |
| 13 | Cyclohexyl isothiocyanate | C7H11NS | 141 | 10.247 |  |
| 14 | 2,5-Dimethylundecane | C13H28 | 184 | 11.002 |  |
| 15 | 2,5-Dimethyl-1-phenyl-1,3-hexanedione | C14H18O2 | 218 | 11.126 |  |
| 16 | 3-Bromodecane | C10H21Br | 220 | 12.024 |  |
| 17 | 2,6-Dimethyl-6-trifluoroacetoxyoctane | C12H21F3O2 | 254 | 12.200 |  |
| 18 | 2,3,6-Trimethyldecane | C13H28 | 184 | 12.229 |  |
| 19 | Dioctanoyl peroxide | C16H30O4 | 286 | 13.311 |  |
| 20 | 4,6-Dimethyldodecane | C14H30 | 198 | 13.566 |  |
| 21 | 2,3-Dimethyldodecane | C14H30 | 198 | 13.833 |  |
| 22 | 3-Methylnonane | C10H22 | 142 | 13.869 |  |
| 23 | Trans-2-nonen-1-ol | C9H18O | 142 | 14.448 |  |
| 24 | 1-Nonadecanol | C19H40O | 284 | 14.460 |  |
| 25 | *1-Undecanol | C11H24O | 172 | 14.612 |  |
| 26 | 2-Methyldecane | C11H24 | 156 | 14.767 |  |
| 27 | 2-Methyleicosane | C21H44 | 296 | 15.026 |  |
| 28 | 3,5-Dimethyloctane | C10H22 | 142 | 15.085 |  |
| 29 | Tridecanal | C13H26O | 198 | 15.102 |  |
| 30 | Dichloroacetic acid, decyl ester | C12H22Cl2O2 | 269 | 15.375 |  |
| 31 | 2,6-Di-tert-butyl-P-benzoquinone | C14H20O2 | 220 | 15.400 |  |
| 32 | 2-Ethyl-1-dodecanol | C14H30O | 214 | 15.957 |  |
| 33 | 1-Iodododecane | C12H25I | 296 | 16.040 |  |
| 34 | 2,2,3,3,5,6,6-Heptamethylheptane | C14H30 | 198 | 16.217 |  |
| 35 | 2,3-Dimethyloctane | C10H22 | 142 | 16.267 |  |
| 36 | Cedran-8-ol | C15H26O | 222 | 16.396 |  |
| 37 | 2-Isopropyl-5-methyl-1-heptanol | C11H24O | 172 | 16.561 |  |
| 38 | 2-Methyltridecane | C14H30 | 198 | 16.767 |  |
| 39 | Trichloroacetic acid dodecyl ester | C14H25Cl3O2 | 331 | 16.887 |  |
| 40 | 2-Hexyldecanol | C16H34O | 242 | 17.287 |  |
| 41 | Benzeneacetic acid, 4-tridecyl ester | C21H34O2 | 318 | 17.632 |  |
| 42 | Decanoic acid, decyl ester | C20H40O2 | 312 | 17.649 |  |
| 43 | 3-Ethyl-4-methylhexane | C9H20 | 128 | 17.715 |  |
| 44 | 3-Methyl-5-propylnonane | C13H28 | 184 | 17.900 |  |
| 45 | Hexanedioic acid, dibutyl ester | C14H26O4 | 258 | 17.975 |  |
| 46 | 3,8-Dimethyldecane | C12H26 | 170 | 18.150 |  |
| 47 | 2,6,10,14-Tetramethylpentadecane | C19H40 | 268 | 18.257 |  |
| 48 | (S)-5-(Isobutyl) imidazolidine-2,4-dione | C7H12N2O2 | 156 | 18.258 |  |
| 49 | Pentadecanal | C15H30O | 226 | 18.376 |  |
| 50 | 1,2-Diphenylcyclobutane | C16H16 | 208 | 18.410 |  |
| 51 | Tetradecanal | C14H28O | 212 | 18.418 |  |
| 52 | Diisononylphthalate | C26H42O4 | 418 | 18.543 |  |
| 53 | 1-Chloroeicosane | C20H41Cl | 317 | 18.775 |  |
| 54 | 2,2,6,6-Tetramethylheptane | C11H24 | 156 | 18.815 |  |
| 55 | 2-Bromododecane | C12H25Br | 249 | 18.868 |  |
| 56 | 2,6,10-Trimethyltetradecane | C17H36 | 240 | 19.030 |  |
| 57 | Tetradecanoic acid, ethyl ester | C16H32O2 | 256 | 19.325 |  |
| 58 | Triacontanoic acid, methyl ester | C31H62O2 | 466 | 19.409 |  |
| 59 | 2,6,10,15-Tetramethylheptadecane | C21H44 | 296 | 19.428 |  |
| 60 | Dodecanoic acid, 2,3-bis(acetyloxy)propyl ester | C19H34O6 | 358 | 19.624 |  |
| 61 | Menthyl salicylate | C17H24O3 | 276 | 19.652 |  |
| 62 | 5-Nonylamine | C9H21N | 143 | 19.764 |  |
| 63 | 2-Ethyl-2-methyltridecanol | C16H34O | 242 | 19.819 |  |
| 64 | 1,2-Benzenedicarboxylic acid, diisodecyl ester | C28H46O4 | 446 | 20.133 |  |
| 65 | 1-Pentadecanol | C15H32O | 228 | 20.239 |  |
| 66 | *1-Eicosanol | C20H42O | 298 | 20.305 |  |
| 67 | Dibutyl phthalate | C16H22O4 | 278 | 20.473 |  |
| 68 | Butyl isodecyl phthalate | C22H34O4 | 362 | 20.484 |  |
| 69 | 3,3,4-Trimethyldecane | C13H28 | 184 | 20.650 |  |
| 70 | *Pentadecanoic acid | C15H30O2 | 242 | 20.716 |  |
| 71 | Hexadecanoic acid, ethyl ester | C18H36O2 | 284 | 21.032 |  |
| 72 | 1,2-Benzenedicarboxylic acid, butyloctyl ester | C20H30O4 | 334 | 21.275 |  |
| 73 | 1-Docosene | C22H44 | 308 | 21.385 |  |
| 74 | Eicosanoic acid, ethyl ester | C22H44O2 | 340 | 21.422 |  |
| 75 | Docosanoic acid, ethyl ester | C24H48O2 | 368 | 21.552 |  |
| 76 | Oleic Acid | C18H34O2 | 282 | 21.706 |  |
| 77 | *Eicosanoic acid | C20H40O2 | 312 | 21.770 |  |
| 78 | 2-Isopropyl-3-(methoxycarbonyl)-1,3-oxazolidine-4-carboxylic acid | C9H15NO5 | 217 | 21.786 |  |
| 79 | *1-Octadecanol | C18H38O | 270 | 22.392 |  |
| 80 | Tetratriacontane | C34H70 | 478 | 22.714 |  |
| 81 | Octadecanoic acid, ethyl ester | C20H40O2 | 312 | 22.802 |  |
| 82 | Ethyl Oleate | C20H38O2 | 310 | 23.017 |  |
| 83 | *Heptadecanoic acid, ethyl ester | C19H38O2 | 298 | 23.151 |  |
| 84 | (E)-9-Octadecenoic acid, ethyl ester | C20H38O2 | 310 | 23.226 |  |
| 85 | Hexadecanoic acid, 2-hydroxyethyl ester | C18H36O3 | 300 | 23.874 |  |
| 86 | Ethyl 9-hexadecenoate | C18H34O2 | 282 | 24.447 |  |
| 87 | 11-Eicosenoic acid, methyl ester | C21H40O2 | 324 | 24.458 |  |
| 88 | Ethyl 2-ethyl-2-tridecanoate | C18H36O2 | 284 | 24.662 |  |
| 89 | 4-t-Butoxy-3-hydroxy-butyric acid, ethyl ester | C10H20O4 | 204 | 24.724 |  |
| 90 | Oxalic acid, decyl 3,5-difluorophenyl ester | C18H24F2O4 | 342 | 24.728 |  |
| 91 | 1,54-Dibromo tetrapentacontane | C54H108Br2 | 914 | 24.902 |  |
| 92 | Mono(2-ethylhexyl) phthalate | C16H22O4 | 278 | 25.212 |  |
| 93 | 1-Iodooctadecane | C18H37I | 380 | 25.309 |  |
| 94 | Floxuridine | C9H11FN2O5 | 246 | 25.465 |  |
| 95 | Ether, isopropyl 2-benzyl-2-propenyl | C13H18O | 190 | 25.985 |  |
| 96 | 3-(2-Carboxy-1-methyl-ethoxy)-5-phenyl-pentanoic acid, rthyl ester | C17H24O5 | 308 | 25.989 |  |
| 97 | Pentanoic acid, 2-methyl, butyl ester | C10H20O2 | 172 | 26.129 |  |
| 98 | Hexanoic acid, heptadecyl ester | C23H46O2 | 354 | 26.208 |  |
| 99 | Oleamide | C18H35NO | 281 | 26.553 |  |
| 100 | Glyceryl monostearate | C21H42O4 | 358 | 26.608 |  |
| 101 | Octadecanoic acid, 2-hydroxyethyl ester | C20H40O3 | 328 | 26.675 |  |
| 102 | (Z)-13-docosenamide | C22H43NO | 337 | 27.452 |  |
| 103 | *Erucic acid, ethyl ester | C24H46O2 | 366 | 27.872 |  |
| 104 | 13,15-Octacosadiyne | C28H50 | 386 | 28.090 |  |
| 105 | Cholesteryl myristate | C41H72O2 | 597 | 28.411 |  |
| 106 | Squalene | C30H50 | 410 | 28.521 |  |
| 107 | Cholest-2-ene | C27H46 | 370 | 29.950 |  |
| 108 | Cholesterol chloroformate | C28H45ClO2 | 448 | 30.121 |  |
| 109 | 15,17,19,21-Hexatriacontatetrayne | C36H58 | 490 | 30.178 |  |
| 110 | 4,6- Cholestadien-3beta-ol | C27H44O | 384 | 30.278 |  |
| 111 | 1-Heptatriacotanol | C37H76O | 536 | 30.817 |  |
| 112 | Cholesterol | C27H46O | 386 | 31.502 |  |
| 113 | Coprosterol | C27H48O | 388 | 33.421 |  |
| 114 | Cholestan-3-ol | C27H48O | 388 | 34.491 |  |
| 115 | 5-Alpha-cholestan-3-one | C27H46O | 386 | 34.526 |  |
| 116 | Beta-Sitosterol | C29H50O | 414 | 36.443 |  |
| 117 | Cholest-5-en-3-ol | C27H46O | 386 | 36.460 |  |
| 118 | Ergosta-5,7-dien-3 beta-ol | C28H46O | 398 | 36.611 |  |
| 119 | Ergosta-8,14-dien-3-ol | C28H46O | 398 | 36.715 |  |
| 120 | Cholest-4-en-3-one | C27H44O | 384 | 37.418 |  |

Table note: ^*^: Compounds identified using authentic standards, 2,5-Dimethylhexane (98%), p-Benzoquinone (99.5%), 1-Undecanol (99.5%), 1-Eicosanol (99.5%), Pentadecanoic acid (99.5%), Eicosanoic acid (99.5%), 1-Octadecanol (99.5%), Heptadecanoic acid ethyl ester (98%), Erucic acid ethyl ester (98%) were purchased from Sigma-Aldrich (Shanghai) trading co. LTD, Shanghai, China.
