# Supplementary material for: Symbiotic microbiota and odor ensure mating in time for giant pandas
Source: Front Microbiol. 2022 Nov 17;13:1015513. doi: 10.3389/fmicb.2022.1015513 (PMC9712809; doi:10.3389/fmicb.2022.1015513)
Supplement: Supplementary file 2 [file Table_2.DOCX]

Table S2. The relative abundance of enriched genera between male and female giant pandas by the LDA analysis

| genus | Relative abundance in  male | | Relative abundance in female | |
| --- | --- | --- | --- | --- |
|  | Mean % | SD % | Mean % | SD % |
| *Arcanobacterium* | 0.1737 | 0.1373 | 0.0517 | 0.0907 |
| *Helicobacter* | 0.0496 | 0.0746 | 0.3118 | 0.1832 |
| *Terrisporobacter* | 0.0522 | 0.0593 | 0.5241 | 0.5427 |
| *Dielma* | 0.0000 | 0.0000 | 0.0111 | 0.0202 |
| *Cellulosilyticum* | 0.0022 | 0.0060 | 0.0080 | 0.0118 |
| *Solirubrobacter* | 0.0000 | 0.0000 | 0.0024 | 0.0030 |
| *Rhizorhapis* | 0.0016 | 0.0027 | 0.0040 | 0.0016 |
| *Weissella* | 0.0006 | 0.0013 | 0.0517 | 0.1013 |
| *Novosphingobium* | 0.0025 | 0.0036 | 0.0103 | 0.0054 |
