# Supplementary material for: Symbiotic microbiota and odor ensure mating in time for giant pandas
Source: Front Microbiol. 2022 Nov 17;13:1015513. doi: 10.3389/fmicb.2022.1015513 (PMC9712809; doi:10.3389/fmicb.2022.1015513)
Supplement: Supplementary file 3 [file Table_3.DOCX]

Table S3. The relative abundance of enriched genera between the prophase, metaphase and anaphase of female giant pandas by the LDA analysis

| genera | Relative abundance in prophase | | Relative abundance in metaphase | | Relative abundance in anaphase | |
| --- | --- | --- | --- | --- | --- | --- |
|  | Mean % | SD % | Mean % | SD % | Mean % | SD % |
| *Moraxella* | 0.0271 | 0.0473 | 0.0042 | 0.0045 | 0.0000 | 0.0000 |
| *Trichococcus* | 0.0305 | 0.0307 | 0.0111 | 0.0099 | 0.0007 | 0.0016 |
| *Carnobacterium* | 0.1381 | 0.1271 | 0.1228 | 0.2533 | 0.0035 | 0.0049 |
| *Microlunatus* | 0.0326 | 0.0445 | 0.0208 | 0.0179 | 0.0049 | 0.0076 |
| *norank Geodermatophilaceae* | 0.1575 | 0.1096 | 0.0326 | 0.0391 | 0.0125 | 0.0226 |
| *Tessaracoccus* | 0.0125 | 0.0126 | 0.0007 | 0.0016 | 0.0000 | 0.0000 |
| *Pseudoclavibacter* | 0.2373 | 0.1818 | 0.2324 | 0.1455 | 0.0409 | 0.0576 |
| *unclassified Dermatophilaceae* | 0.1637 | 0.2094 | 0.0104 | 0.0127 | 0.0014 | 0.0031 |
| *unclassified Propionibacteriaceae* | 0.0791 | 0.0671 | 0.0153 | 0.0223 | 0.0014 | 0.0031 |
| *Rubrobacter* | 0.0146 | 0.0148 | 0.0028 | 0.0062 | 0.0000 | 0.0000 |
| *Terrabacter* | 0.4343 | 0.3090 | 0.1110 | 0.1131 | 0.0715 | 0.1559 |
| *Mucilaginibacter* | 0.0042 | 0.0093 | 0.0000 | 0.0000 | 0.0000 | 0.0000 |
| *Craurococcus* | 0.0069 | 0.0081 | 0.0000 | 0.0000 | 0.0021 | 0.0047 |
| *Janibacter* | 0.9788 | 0.7483 | 0.4599 | 0.6127 | 0.1006 | 0.1788 |
| *Gordonia* | 0.0506 | 0.0958 | 0.0042 | 0.0038 | 0.0014 | 0.0019 |
| *Cellulosilyticum* | 0.0000 | 0.0000 | 0.0007 | 0.0016 | 0.0000 | 0.0000 |
| *Moheibacter* | 0.0069 | 0.0118 | 0.0000 | 0.0000 | 0.0000 | 0.0000 |
| *Jeotgalicoccus* | 0.0125 | 0.0226 | 0.0000 | 0.0000 | 0.0000 | 0.0000 |
| *Luteococcus* | 0.0021 | 0.0019 | 0.0000 | 0.0000 | 0.0000 | 0.0000 |
| *Dietzia* | 0.0250 | 0.0520 | 0.0000 | 0.0000 | 0.0007 | 0.0016 |
| *Staphylococcus* | 0.0243 | 0.0272 | 0.0000 | 0.0000 | 0.0000 | 0.0000 |
| *Brachybacterium* | 6.1602 | 4.3376 | 3.3514 | 3.7293 | 0.7236 | 1.1475 |
| *Brevibacterium* | 7.5616 | 10.6713 | 1.5144 | 2.6093 | 0.0721 | 0.0926 |
| *Kocuria* | 1.1169 | 1.1838 | 0.2601 | 0.2889 | 0.0610 | 0.1181 |
| *Methylobacterium* | 0.0319 | 0.0277 | 0.0444 | 0.0652 | 0.0028 | 0.0045 |
| *Planomicrobium* | 0.0139 | 0.0157 | 0.1027 | 0.1600 | 0.0000 | 0.0000 |
| *Anaerococcus* | 0.0950 | 0.1469 | 0.2400 | 0.2072 | 0.2407 | 0.2720 |
| *Bifidobacterium* | 0.0007 | 0.0016 | 0.0035 | 0.0078 | 0.0000 | 0.0000 |
| *Enterococcus* | 0.1700 | 0.2466 | 0.5897 | 1.0311 | 0.0173 | 0.0274 |
| *Actinomyces* | 0.0000 | 0.0000 | 0.0007 | 0.0016 | 0.0000 | 0.0000 |
| *Pontibacter* | 0.0007 | 0.0016 | 0.0000 | 0.0000 | 0.0000 | 0.0000 |
| *Terriglobus* | 0.0007 | 0.0016 | 0.0055 | 0.0053 | 0.0000 | 0.0000 |
| *Campylobacter* | 0.0583 | 0.1110 | 0.4724 | 0.9198 | 3.8203 | 6.4528 |
| *unclassified Pseudomonadaceae* | 0.7333 | 1.2042 | 2.7693 | 3.4903 | 8.2352 | 6.3950 |
