# Supplementary material for: Symbiotic microbiota and odor ensure mating in time for giant pandas
Source: Front Microbiol. 2022 Nov 17;13:1015513. doi: 10.3389/fmicb.2022.1015513 (PMC9712809; doi:10.3389/fmicb.2022.1015513)
Supplement: Supplementary file 4 [file Table_4.DOCX]

Table S4. The information of samples and individuals

| Sample ID | Panda ID | Sex | Age | Family No. | Group |
| --- | --- | --- | --- | --- | --- |
| AGSmale1 | Fufu | Male | 18 | 532 | AGS-male |
| AGSmale2 | Yingying | Male | 11 | 724 | AGS-male |
| AGSmale3 | Abao | Male | 12 | 703 | AGS-male |
| AGSmale4 | Gongzai | Male | 11 | 711 | AGS-male |
| AGSfemale1 | Yazai | Female | 13 | 637 | AGS-female |
| AGSfemale2 | Xingrong | Female | 12 | 680 | AGS-female |
| AGSfemale3 | Jinngjing | Female | 14 | 598 | AGS-female |
| AGSfemale4 | Nini | Female | 11 | 725 | AGS-female |
| AGSfemale5 | Erqiao | Female | 8 | 823 | AGS-female |
| AGSfemale6 | Meilun | Female | 6 | 870 | AGS-female |
| AGSfemale7 | Meibao | Female | 9 | 801 | AGS-female |
| AGSfemale8 | Chengda | Female | 8 | 824 | AGS-female |
| AGSfemale9 | Chenggong | Female | 19 | 522 | AGS-female |
| AGSfemale10 | Dajiao | Female | 13 | 645 | AGS-female |
| AGMfemale1-p | Yazai | Female | 13 | 637 | AGM-prophase |
| AGMfemale2-p | Xingrong | Female | 12 | 680 | AGM-prophase |
| AGMfemale3-p | Jinngjing | Female | 14 | 598 | AGM-prophase |
| AGMfemale4-p | Meibao | Female | 9 | 801 | AGM-prophase |
| AGMfemale5-p | Dajiao | Female | 13 | 645 | AGM-prophase |
| AGMfemale1-m | Yazai | Female | 13 | 637 | AGM-metaphase |
| AGMfemale2-m | Xingrong | Female | 12 | 680 | AGM-metaphase |
| AGMfemale3-m | Jinngjing | Female | 14 | 598 | AGM-metaphase |
| AGMfemale4-m | Meibao | Female | 9 | 801 | AGM-metaphase |
| AGMfemale5-m | Dajiao | Female | 13 | 645 | AGM-metaphase |
| AGMfemale1-a | Yazai | Female | 13 | 637 | AGM-anaphase |
| AGMfemale2-a | Xingrong | Female | 12 | 680 | AGM-anaphase |
| AGMfemale3-a | Jinngjing | Female | 14 | 598 | AGM-anaphase |
| AGMfemale4-a | Meibao | Female | 9 | 801 | AGM-anaphase |
| AGMfemale5-a | Dajiao | Female | 13 | 645 | AGM-anaphase |
