# Supplementary figures and images for: Symbiotic microbiota and odor ensure mating in time for giant pandas
Source: Front Microbiol. 2022 Nov 17;13:1015513. doi: 10.3389/fmicb.2022.1015513 (PMC9712809; doi:10.3389/fmicb.2022.1015513)

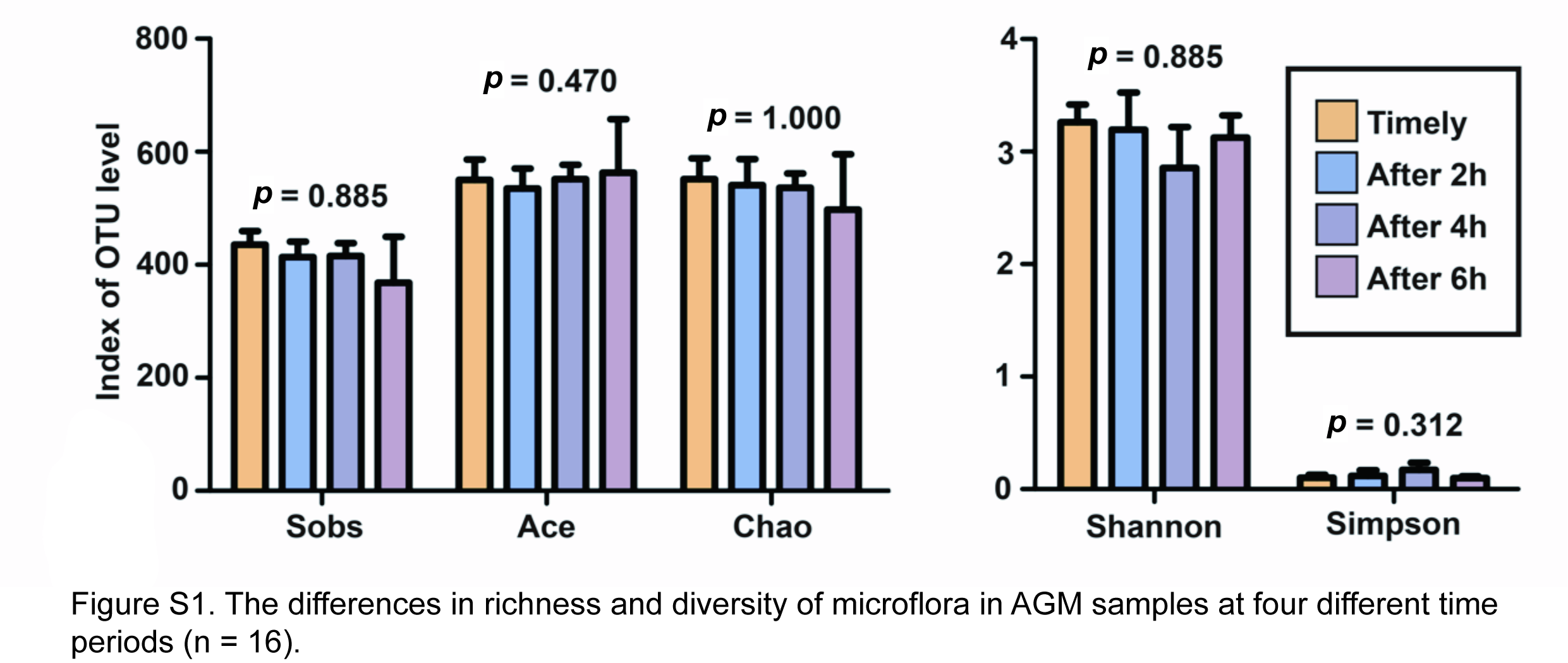

Supplement: Supplementary file 5 [file Image_1.TIF]

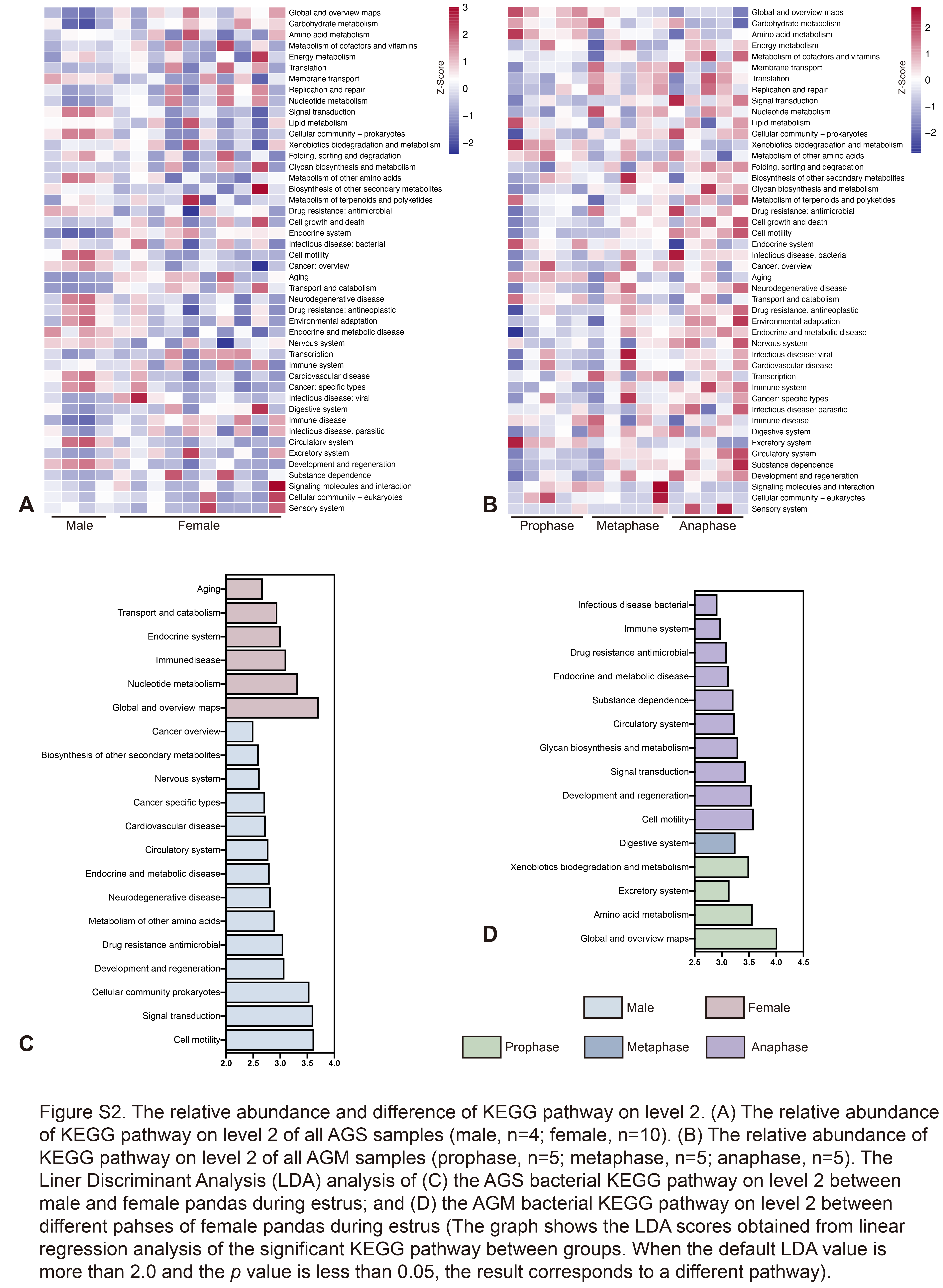

Supplement: Supplementary file 6 [file Image_2.TIF]

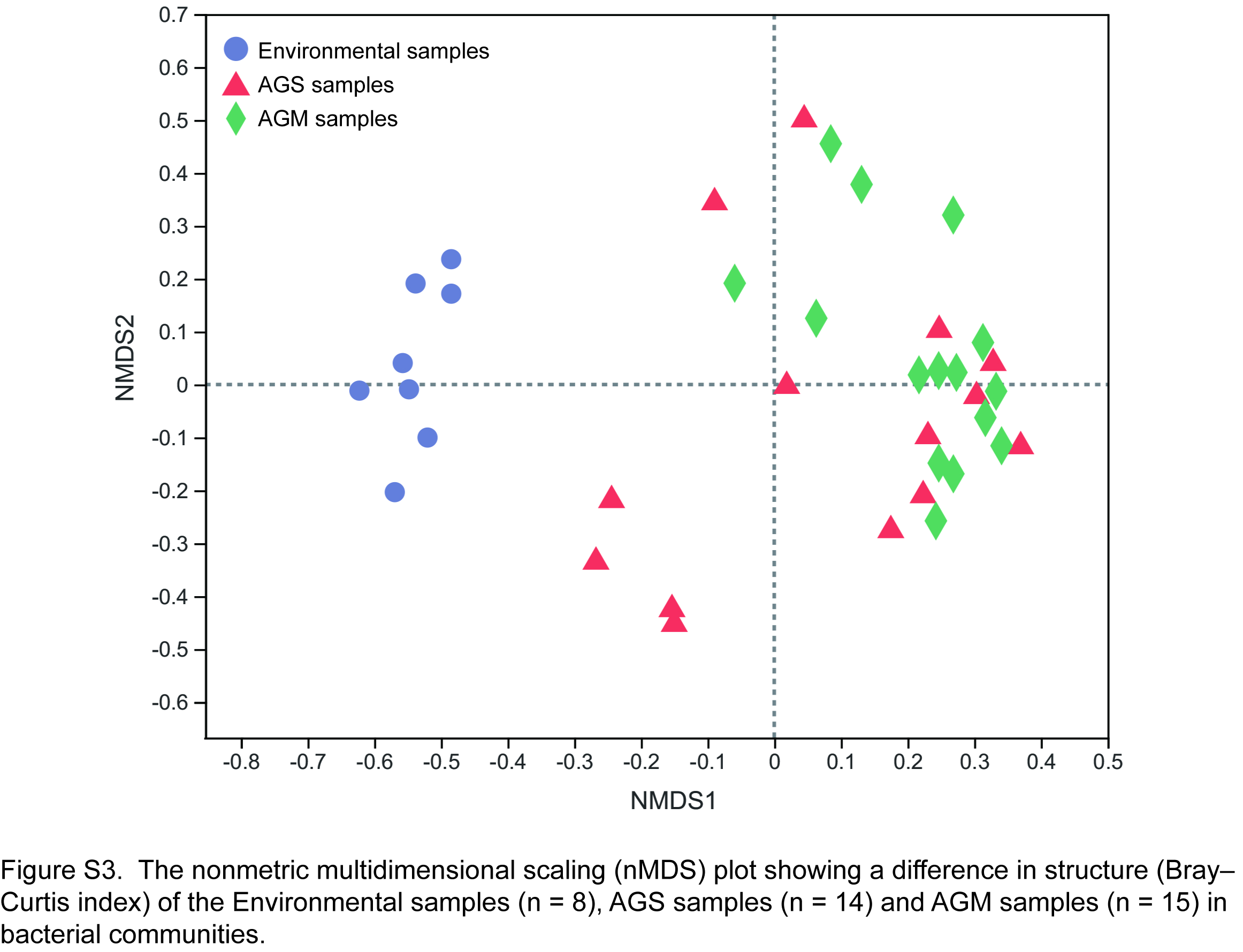

Supplement: Supplementary file 7 [file Image_3.TIF]
